# Supplementary figures and images for: Slipknotted and unknotted monovalent cation-proton antiporters evolved from a common ancestor
Source: PLoS Comput Biol. 2021 Oct 14;17(10):e1009502. doi: 10.1371/journal.pcbi.1009502 (PMC8562792; doi:10.1371/journal.pcbi.1009502)

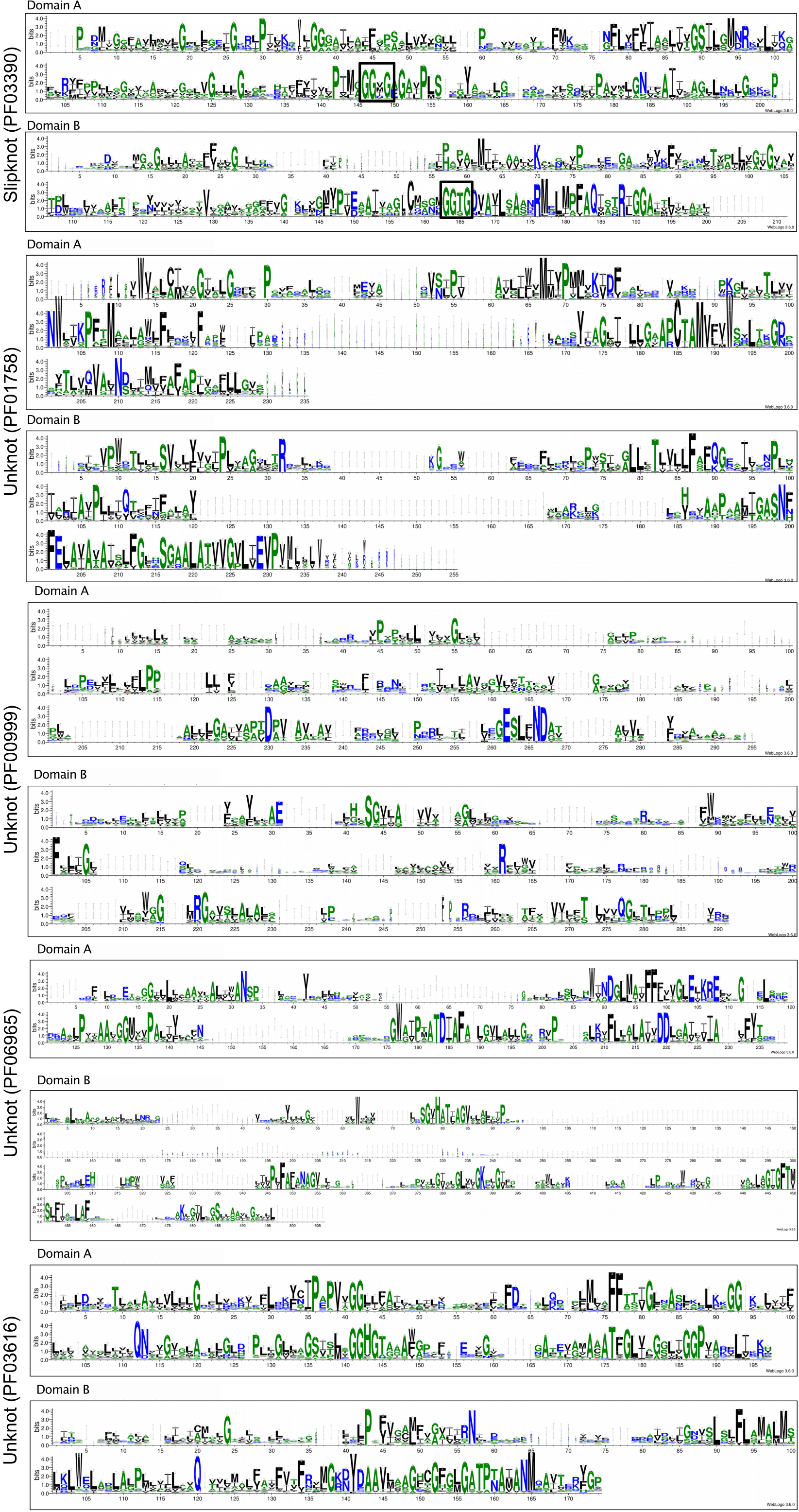

Supplement: S1 Fig — Sequence logo of slipknot family PF03390 showing that multiple glycines are highly conserved across the whole family. The logos were generated from families multiple sequence alignments (available in Pfam) with WebLogo3. (TIFF) [file pcbi.1009502.s001.tiff]

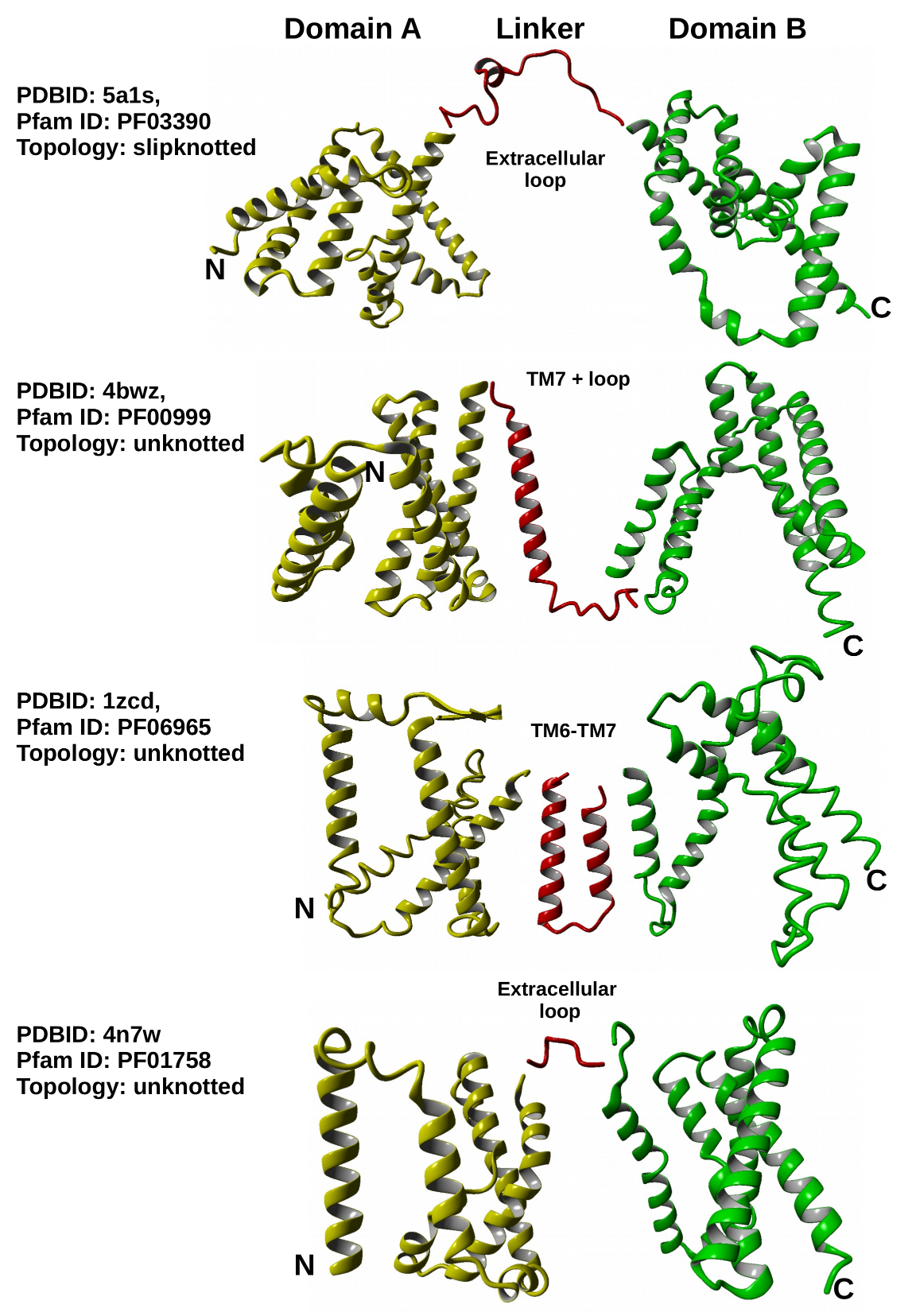

Supplement: S2 Fig — Figure shows that slipknotted and unknotted proteins are composed of two inverted domains which are connected by the linker. Panel A shows slipknotted structure (PDBID: 5a1s). From left to right: domain A, linker and domain B are shown. Similarly, panel B-C shows the linkers between the domains in unknotted structures. (TIFF) [file pcbi.1009502.s002.tiff]

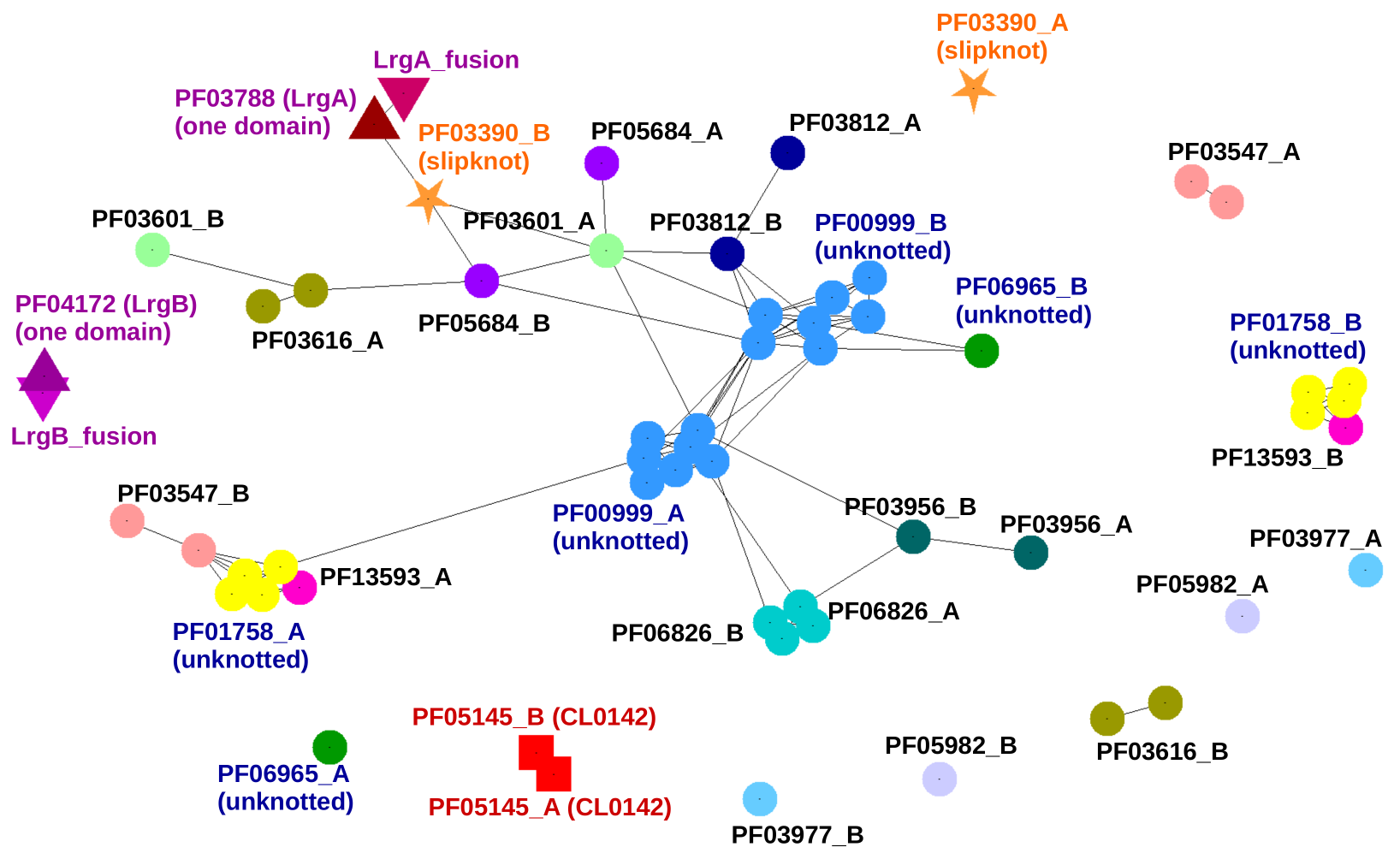

Supplement: S3 Fig — Comparison of domains profiles, shown at cut-off 1e-5. Every domain profile is shown as one point with different shapes: star, circle, triangle, square. The connections between domains are shown as straight lines. The connection indicates that profile-profile alignment of these domains has significance value 1e-5 or less. Every family is colored in unique color, same as in the main Fig 5A. Two domains of the slipknotted family are shown as orange stars. All families from CL0062 are shown as circles colored according to the family. The families IDs with known unknotted topology are highlighted in blue font. The one-domain proteins (PF03788 and PF04172) are shown as triangles and families IDs are colored in magenta font. Two domains of PF05145 (CL0142) are shown as red squares. Families (PF00999 (blue), PF01758 (yellow), PF3547 (pink), PF03616 (olive), PF06826 (dark cyan) were divided into several subgroups based on full sequence clustering (S10 Fig), therefore there are more than two domains in these families. Domain A and B of PF00999 have separated into two clear clusters. (TIFF) [file pcbi.1009502.s003.tiff]

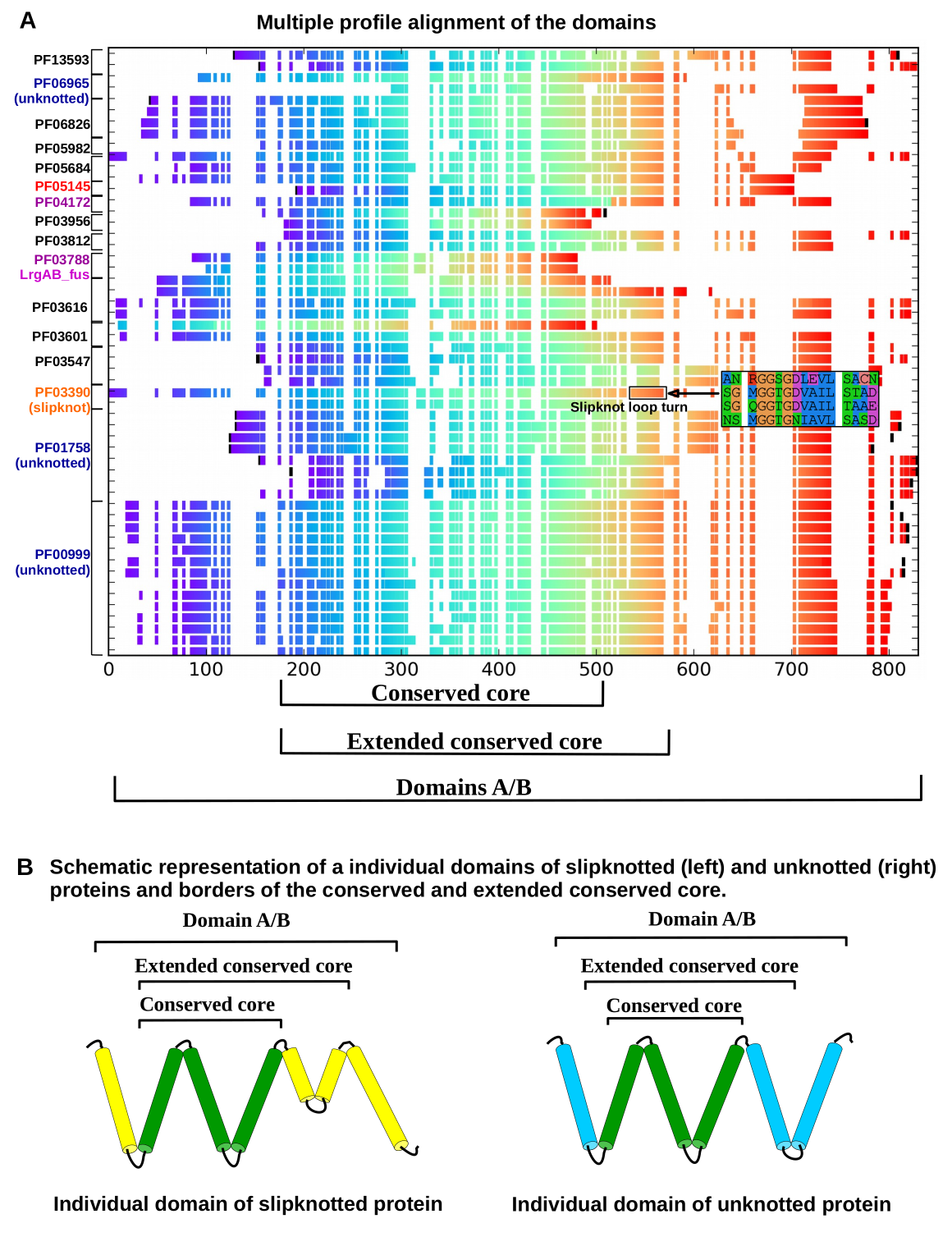

Supplement: S4 Fig — (A) Multiple alignment of domains profiles revealed the conserved core region. Y-axis lists all families which pass through the alignment threshold 1e-3. Every line in the alignment represents family profile. On the X-axis sequence length colored from blue to red. White spaces in the alignment are present when no significant similarity was found between the profiles. The borders of the domain and conserved 3 TM helical core are indicated below the plot. The turn in TM12 forming the slipknot loop is highlighted by black rectangle and next to it the multiple sequence alignment with conserved GGxG region is shown. (B) Schematic representation of individual domains of slipknotted and unknotted proteins. (TIFF) [file pcbi.1009502.s004.tiff]

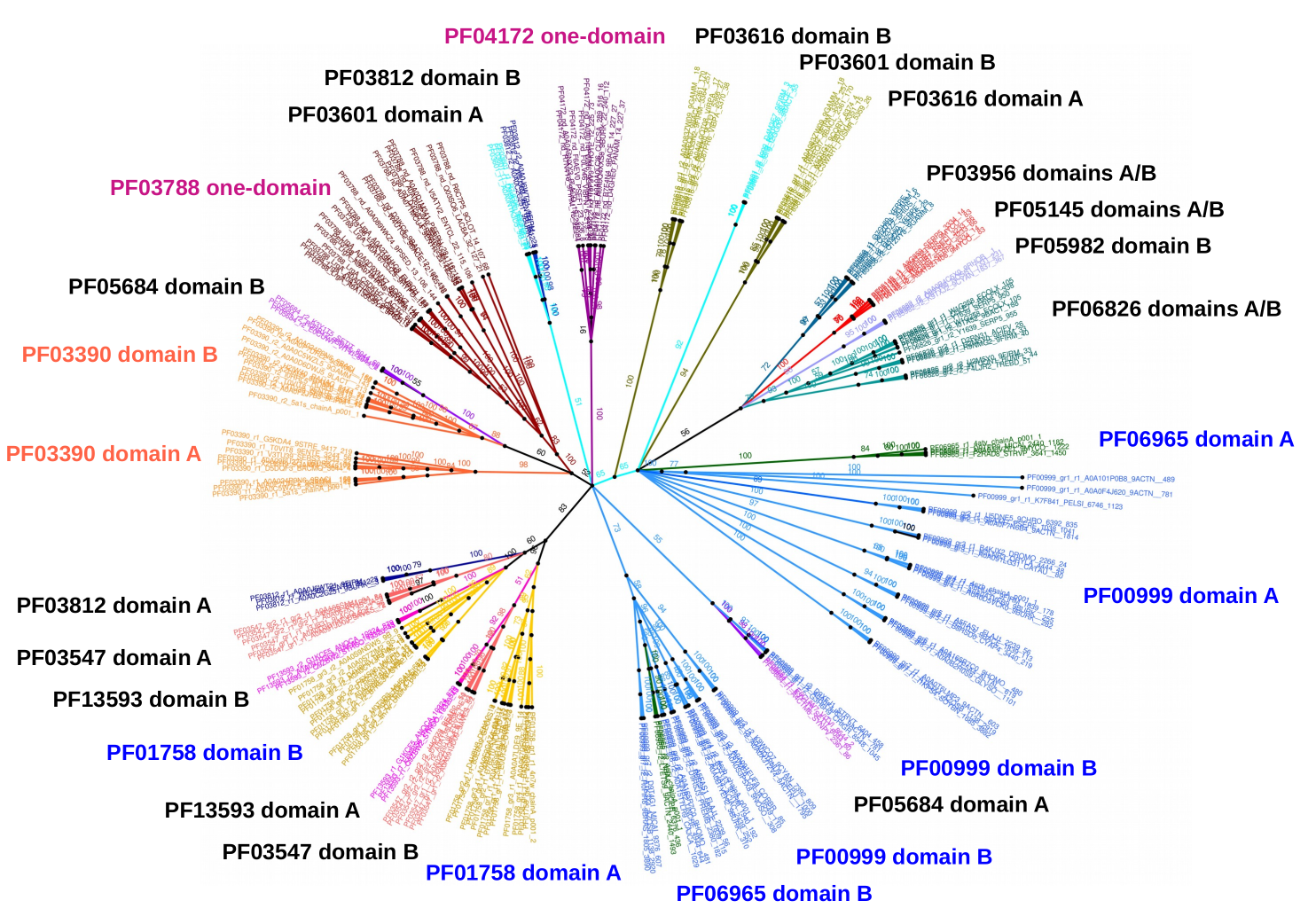

Supplement: S5 Fig — The tree was generated from multiple sequence alignment of the domains using the characteristics matrix multiplied 10 times. The characteristics matrix (S11 Fig) was generated based on profile-profile connections (S3 Fig). For the tree calculation three representative sequences of each family were used. The tree shows several main branches: 1) Both domains of the slipknotted family PF03390 and one-domain family PF03788 are located on the same branch; 2) Another separated branch joins closely related families PF01758, PF013593 and PF3547; 3) Domains A and B of the unknotted family PF00999 were separated into two branches. Domains B of PF00999 are grouped together with the domain B of unknotted family PF06965 and with domain A of PF05684 (unknown topology). Domain A and B of families PF03956, PF06826, PF05145, domain B of PF05982 are located in one branch. (TIFF) [file pcbi.1009502.s005.tiff]

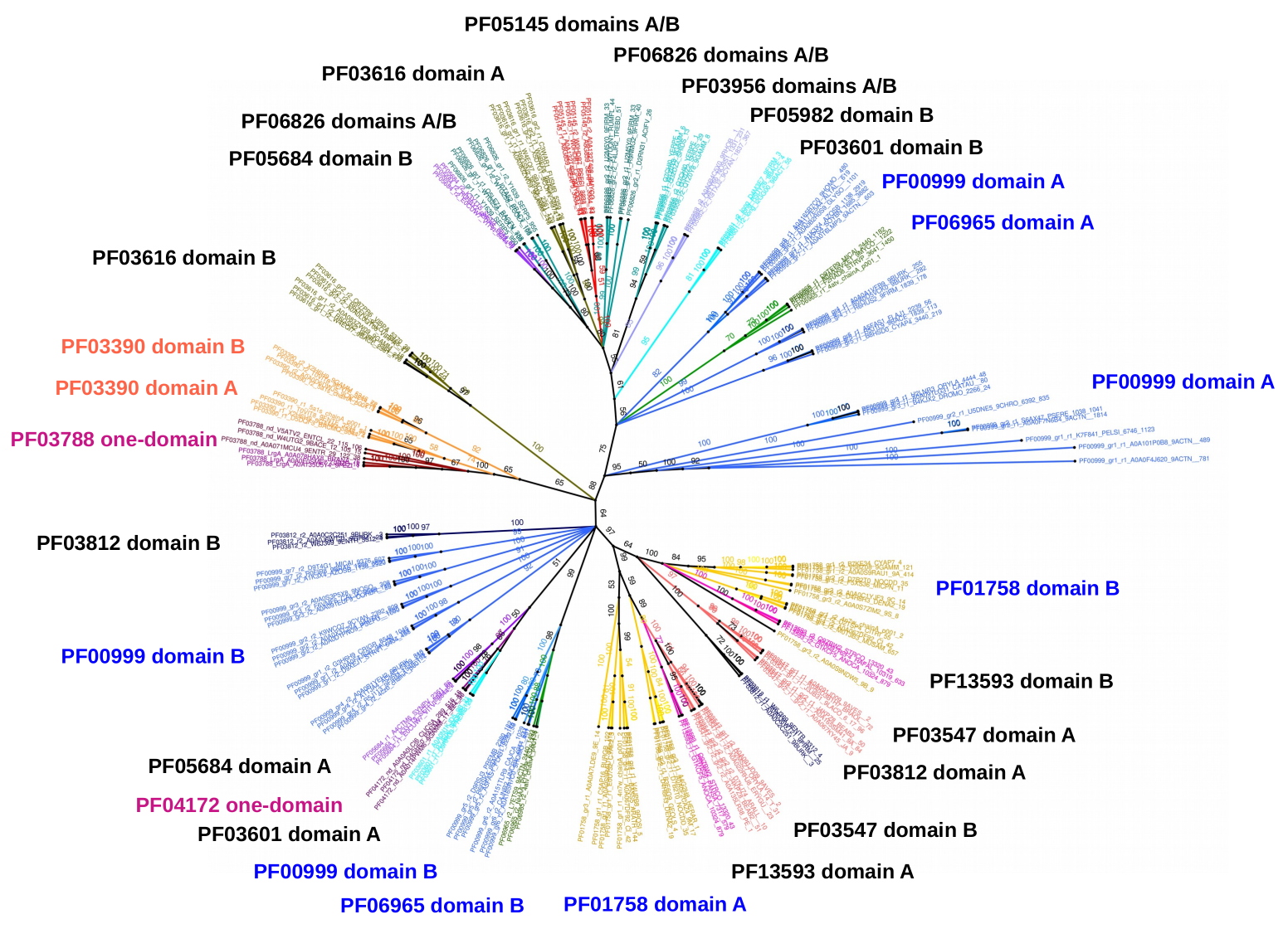

Supplement: S6 Fig — The tree was generated from multiple sequence alignment of the domains using the characteristics matrix multiplied 5 times. The characteristics matrix was generated based on profile-profile connections (Fig 5A and S3 Fig). The characteristics matrix is shown on S11 Fig. For the tree calculation 10 representative sequences of slipknotted PF03390 and one-domain families (PF03788, PF04172) and three representative sequences of remaining families were used. The tree shows three main branches: 1) domains A and B of slipknotted family PF03390 and one-domain family PF03788 are located together on one branch; 2) Another separated branch joins closely related families (domains A and B) PF01758, PF013593 and PF3547; 3) Domains A and B of the unknotted family PF00999 are located on different branches. Domain A of another unknotted family PF06965 is located together with domains A of PF00999 and domain B of PF06965 is placed together with domain B of PF00999. (TIFF) [file pcbi.1009502.s006.tiff]

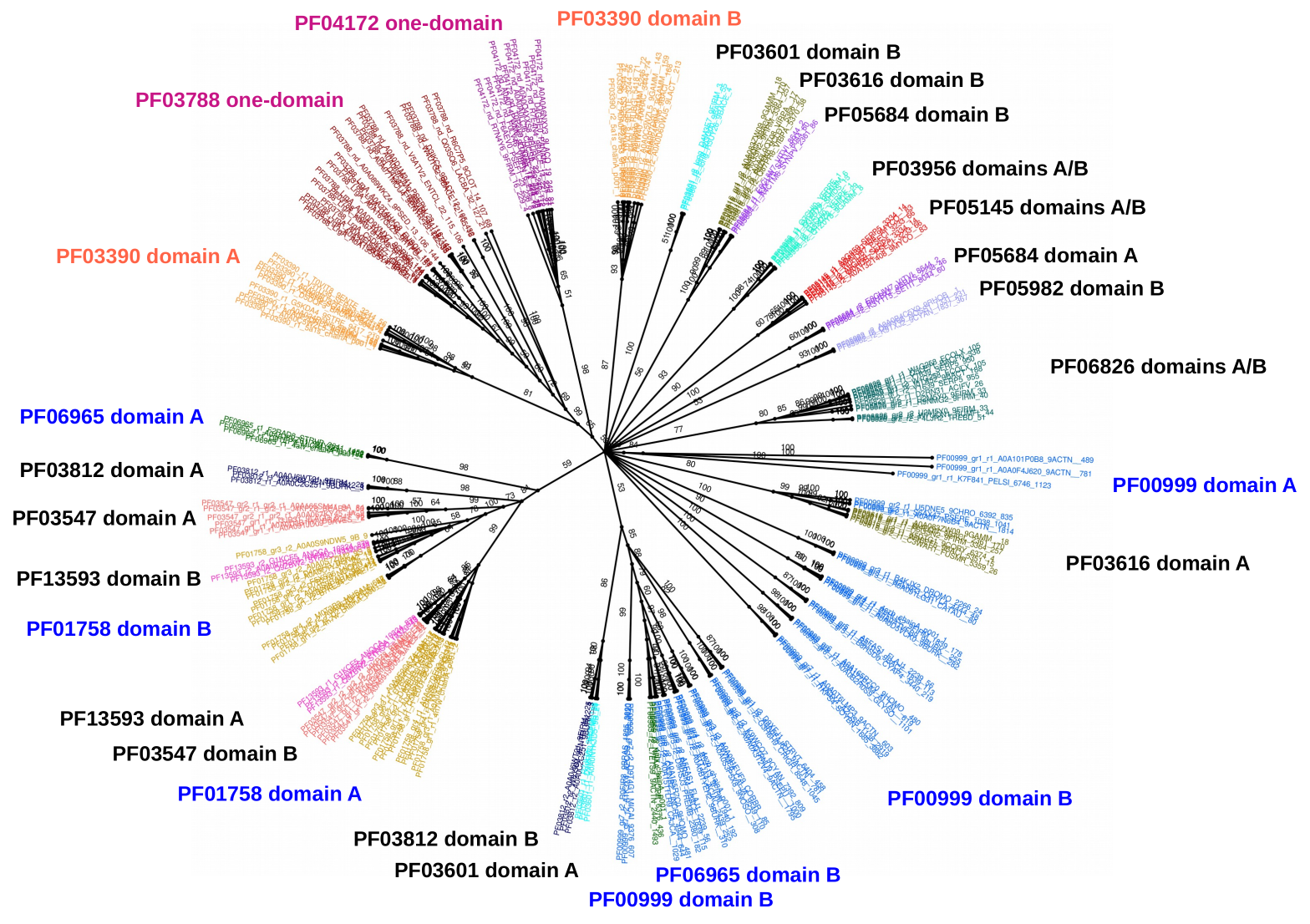

Supplement: S7 Fig — The tree was generated from multiple sequence alignment of the extended conserved core (S4 Fig) which includes the conserved 3-TM helical core + 1 next TM helix (hairpin in slipknotted family). The characteristics matrix based on the profile-profile connections was used as in trees S5 and S6 Figs. Additionally, removed N- and C-terminal regions of the domains were introduced into tree calculation as N/C matrices. Characteristics matrix and N/C matrices were multiplied 10 times. For the tree calculation 10 representative sequences of slipknotted PF03390 and one-domain families (PF03788, PF04172) and three representative sequences of remaining families were used. The tree shows three main branches: 1) slipknotted family PF03390 is placed together with both one-domain families PF03788 and PF04172; 2) Also, as previously, a separated branch joins closely related families PF01758, PF013593 and PF3547; 3) Domains A and B of the unknotted family PF00999 are separated on the tree. However, evolution of other families is not resolved in this tree. PF04172 is placed on the same branch with PF03788 and according to profile analysis these families are distantly related. Also, PF03812(B) and PF03601(A) are together with PF00999(B) which is also in agreement with profile analysis. (TIFF) [file pcbi.1009502.s007.tiff]

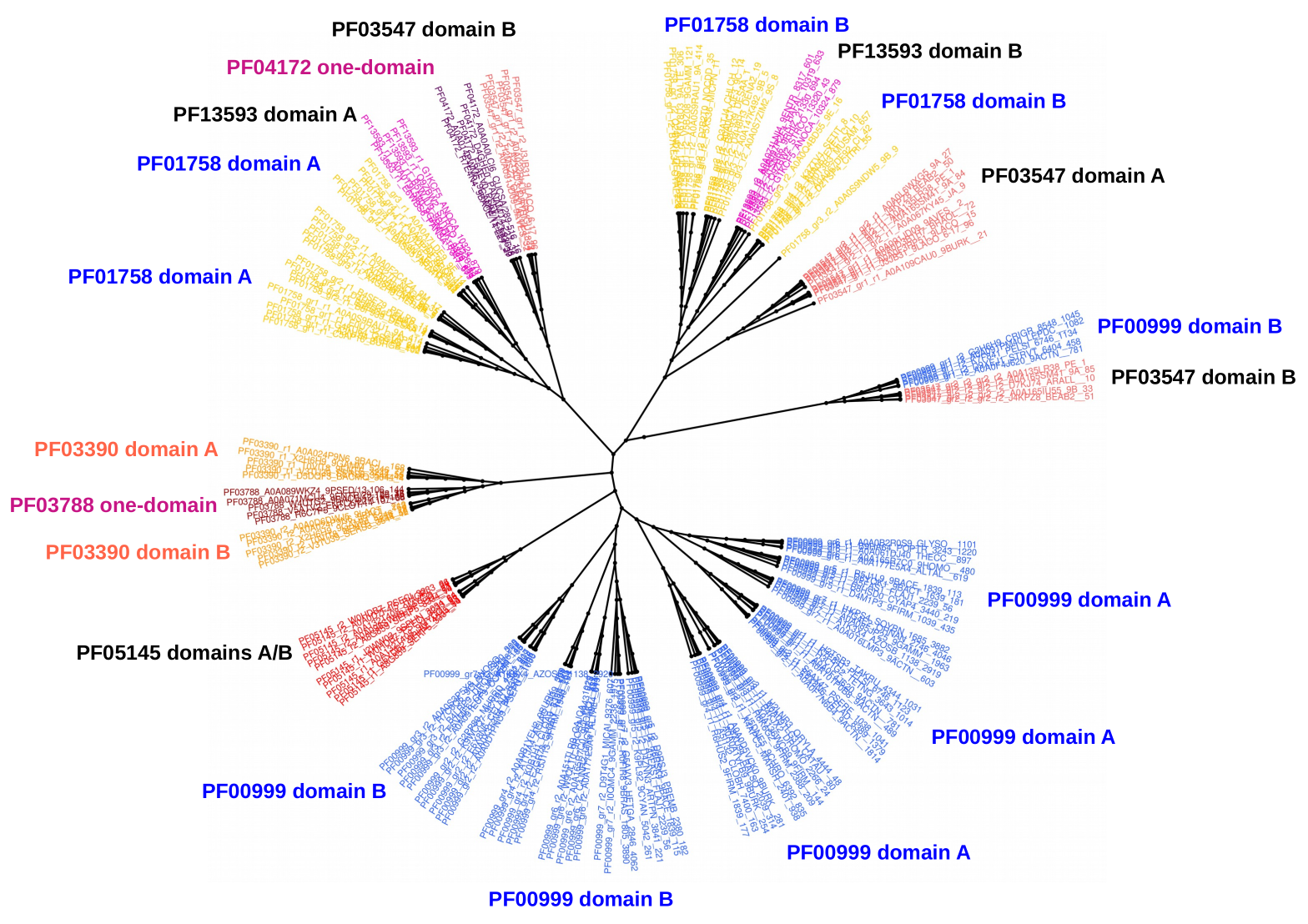

Supplement: S8 Fig — The parsimony tree was generated from the extended core region (conserved core + 1 next TM helix (hairpin in slipknotted family)) same as in the Bayesian phylogenetic tree (S7 Fig). Eight families were used for this analysis: PF00999 (unknotted), PF01758 (unknotted), PF03547 (unknown topology), PF13593 (unknown topology), PF03390 (slipknotted), PF03788 (one-domain), PF04172 (one-domain) and two-domain family PF05145 from the clan CL0142. Five representative sequences of all eight families were used for tree construction. The tree shows four main branches with duplication events producing families: 1) slipknotted family PF03390 and one-domain family PF03788; 2) two-domain closely related families PF01758, PF013593 and PF3547; 3) unknotted PF00999 with two subbranches separating domains A and B; 4) two-domain family PF05145 from another clan (CL0142) was placed on the separate branch. In this tree one-domain family PF04172 is placed together with families PF03547, PF1758 and PF13593 that is in agreement with our profile analysis. The connections between these families are found at e-value 1e-3. (TIFF) [file pcbi.1009502.s008.tiff]

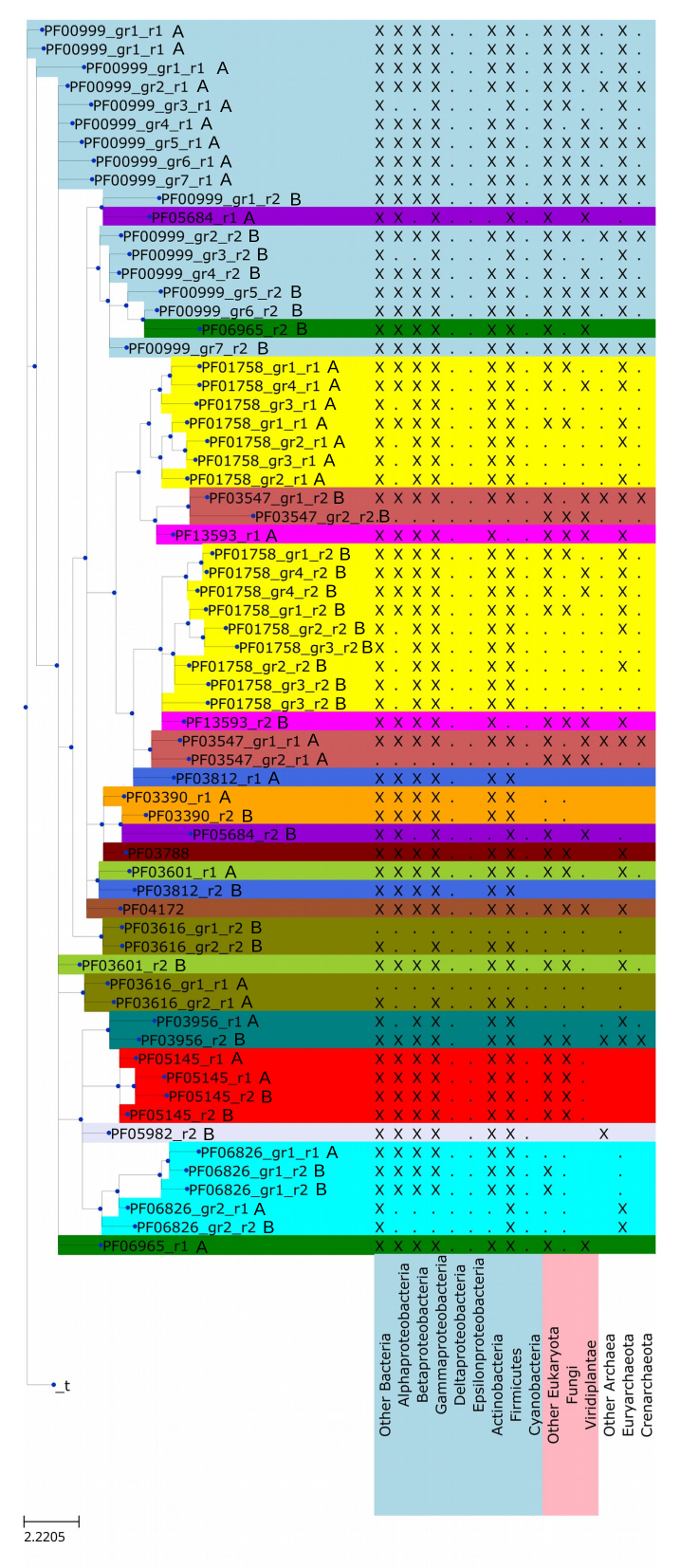

Supplement: S9 Fig — The phylogenetic tree (Fig 5B) is projected on the Tree of Life. Each protein family is colored by unique color similarly as in CLANS (Fig 5A). Presence of the protein family in a particular group of organism is shown by a cross sign “X”. (TIFF) [file pcbi.1009502.s009.tiff]

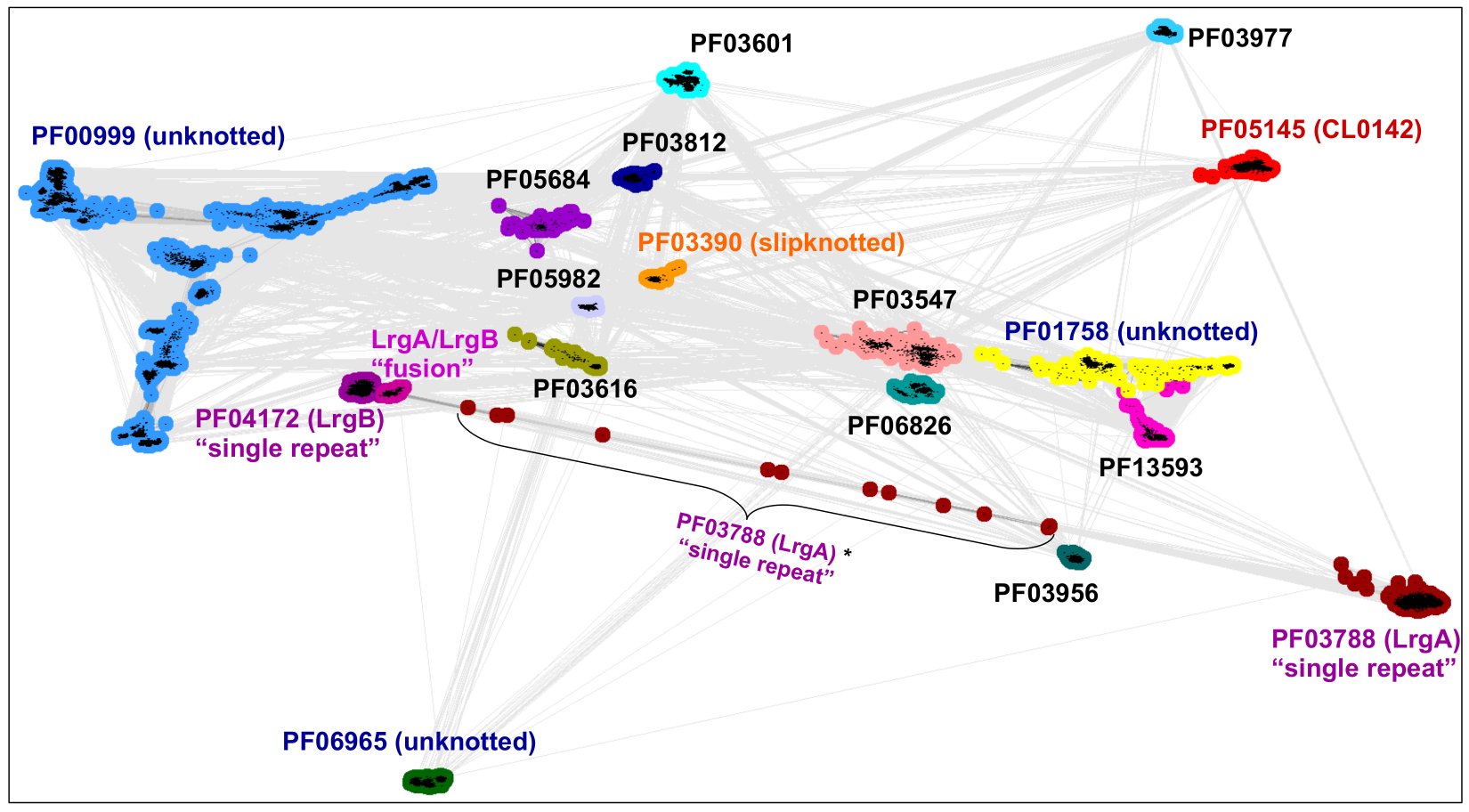

Supplement: S10 Fig — Full set of unaligned sequences of all 17 families were submitted into CLANS all-against-all BLAST search. A single sequence is represented by one “dot”, circle. The sequences clustered in 3D based on pairwise similarity. Mostly, sequences clustered well into families similarly as was assigned by Pfam. Each Pfam family is shown in unique color. The same colors are used in profile-profile analysis (Fig 5A and S3 Fig). The largest (and also unknotted) family PF00999 was clustered into seven groups, all groups colored in blue. The sequences of slipknotted family PF03390 (colored in orange) clustered into one group. The families IDs with unknotted topologies are highlighted with blue font (PF00999, PF06965, PF01758). The family PF05145 from the clan CL0142 is colored red. The one-domain families IDs (LrgA—PF03788, LrgB—PF04172) are highlighted with magenta font. The fusion LrgA/LrgB that partially co-localize with PF04172 (LrgB) is shown in magenta. Some of one-domain LrgA (PF03788) sequences are located in between fusion protein and LrgA, marked with the star sign “*”. (TIFF) [file pcbi.1009502.s010.tiff]

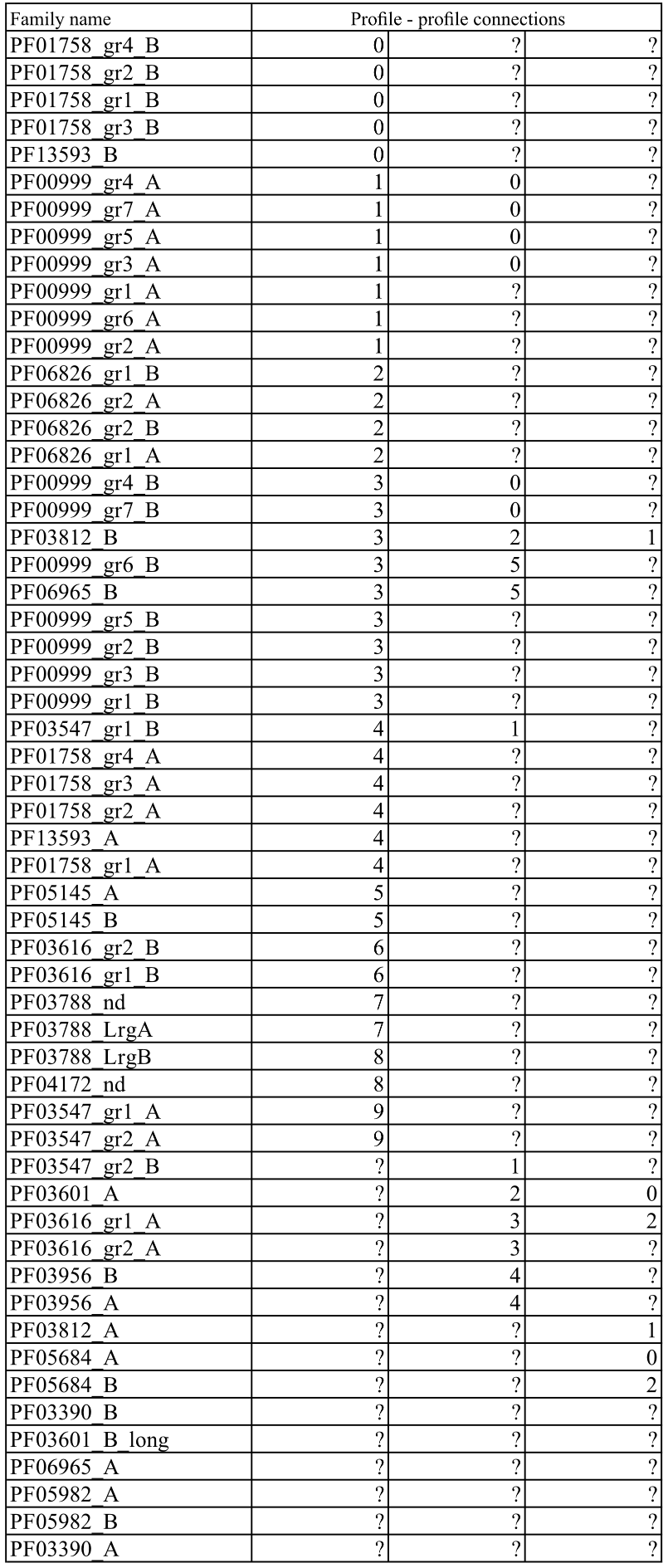

Supplement: S11 Fig — Characteristics matrix used for Bayesian phylogenetic trees generations. The matrix was generated based on the profile-profile connections at the lowest cut-off 1e-5 (Fig 5A and S3 Fig). (TIFF) [file pcbi.1009502.s011.tiff]
