# Supplementary material for: Slipknotted and unknotted monovalent cation-proton antiporters evolved from a common ancestor
Source: PLoS Comput Biol. 2021 Oct 14;17(10):e1009502. doi: 10.1371/journal.pcbi.1009502 (PMC8562792; doi:10.1371/journal.pcbi.1009502)
Supplement: S1 File — Sequence search. Sequence analysis. Procedure to identify the domains. Sequence profiles. Multiple sequence alignment. Phylogeny tree reconstruction. Visualization and figures preparation. (PDF) [file pcbi.1009502.s012.pdf]

Slipknotted and unknotted monovalent cation-proton antiporters evolved from a common ancestor

Supplementary Material

Vasilina Zayats, Agata P. Perlinska, Aleksandra I. Jarmolinska, Borys Jastrzebski, Stanislaw Dunin-Horkawicz and Joanna I. Sulkowska

1 Methods

1.1 Sequence search

We used JackHmmer (hmmer.org) to find protein families distantly related to 2HCT (slipknot) [1], PF00999 (unknotted), PF06965 (unknotted) [20]. We found 13 families which belong to the Pfam clan CL0064. They are all secondary active transporters, which catalyze sodium-proton, aspartate-alanine exchange, sodium-glutamate and Na^+/bile acid symport, etc [21]. Only three of them have solved structures: PF00999, PF06965 and PF01758.
The analysis revealed that AbrB protein family (PF05145, from the clan CL0142) is also related to our target proteins. It is a membrane protein with a different function, and no structures are available for this clan.
Additionally, we found short-length protein families: LrgA (PF03788) and LrgB (PF04172), which do not belong to any clan. In Pfam LrgA and LrgB are indicated to be related to each other and to 2HCT (PF03390), the slipknotted protein. Both are predicted transmembrane proteins, which are involved in cell wall hydrolysis [2, 22]. LrgA is a predicted antiholin-like protein [23], LrgB is co-expressed with LrgA and functions as a transporter [24]. Interestingly, LrgA and LrgB exist in a fused form in plants [25]. Therefore, we also included into our analysis their fused form.
Altogether, we collected 17 protein families and the fusion variant of LrgA-LrgB. Our dataset is composed of: PF03390 (slipknotted, two-domain), 13 (two-domain) families from the CPA_AT clan (CL0064: unknotted PF00999, PF06965 and PF01758; families of unknown stucture/topology: PF03547, PF03601, PF03812, PF03977, PF03956, PF05684, PF05982, PF03616, PF13593, PF06826), PF05145 from CL0142 (structure unknown), and two families with only one-domain (repeat) – LrgA (PF03788) and LrgB (PF04172) (structure unknown).
Most of the sequences were taken from Pfam [11]. However, for several families (PF03547, PF01758, PF6826, PF03956) domain borders were not defined correctly in Pfam (family domain was represented by only one of the repeated domains in PF03547 and PF06826; in PF01758 only middle part of the sequence was present which contained only part of repeated domains A and B). Therefore for these families the full-length sequences were downloaded from UniProt [7]. Then we identified transmembrane domains and extracted the region which contained full A and B domains.

1.2 Sequence analysis

All sequences in the data set were filtered at 90% similarity [12], and only sequences (for two-domain proteins) with lengths deviating by at most half from the average length for a given group were kept. We used CLANS blast to analyze how the sequences are distributed within the dataset [10] (see S10 Fig). The sequences were clustered based on pairwise sequence alignment. We collected all the well-defined groups generated by CLANS blast. The input dataset contained 17 Pfam families, after the CLANS clustering sequences separated into more than 30 groups.
In detail, most of the sequences clustered well into the original families as defined by Pfam. However, sequences of some families were clustered into several subgroups. For example, sequences of PF00999 (the largest and the most diverse family in our dataset) were clustered into seven large subgroups. Similarly, other large families (PF03547, PF01758, PF13593) were also clustered into several subgroups. However, many sequences of the PF13593 family were mixed in together with family PF01758, which indicates that those proteins could be closely related. These are SBF-like (PF13593) and SBF (PF01758) proteins, both characterized as sodium/bile acid co-transporters [26, 27].
Sequences of the non-duplicated LrgA and LrgB clustered separately into two groups. However, the fused LrgA-LrgB version clustered very closely to LrgB. Indeed, LrgB part of the fused sequence remained much more similar to its unfused counterpart, than the LrgA. Sequences of all the other families, including the slipknotted protein (2HCT, PF03390, orange in S10 Fig), clustered into well-defined groups. We extracted sequences of all-well defined clusters (groups or subgroups) into separate groups and named them as Pfam family ID + group (gr) identified by CLANS, for ex. PF00999_gr1, PF00999_gr2, etc.
For every group we generated a multiple sequence alignment with PROMALS3D [5].

1.3 Procedure to identify the domains

Then, for every separate group (out of 30 given by the previous step) we identified the repeated domains. First, we tried several webservers to identify the domains, however, for most of our families no repeated domains could be found. To overcome this, we run HHpred [3] prediction for each family against Pfam and PDB databases [13]. For each query sequence the most similar structure (amongst the 4 structures of proteins from our dataset available in PDB) was identified, and used as a guide to extract the sequences of the repeated domains in all groups. Additionally, transmembrane regions were predicted with MINNOU [4]. Then, sequences were aligned to the most similar structure. The sequences were divided into two domains (A and B) manually in BioEdit [17]. Finally, we run CLANS with Blast on sequences of the separated/individual domains to analyze the new dataset.

1.4 Sequence profiles

After all the groups were separated into two domains (A and B), we generated sequence profiles for each domain using HHmake. Next, we compared all the families domain profiles. The profile comparison was done using HHsearch as one-vs-all comparison for each domain profile [19]. Another round of CLANS clustering was performed based on the results of this comparison (Figs 5A and S3).

1.5 Multiple sequence alignment

Multiple sequence alignment was built by replacing profile states in a multiple profile alignment (MPA) with corresponding sequence residues (S4 Fig). MPA, in turn, was created by maximizing the agreement between the multialignment and the pairwise profile-profile alignments generated by HHsearch. The in-house algorithm used follows the principles of the maximum weight trace alignment, and is available online at https://github.com/ilbsm/HHsearch-results-aligner.

1.6 Phylogeny tree reconstruction

The inputs to MrBayes had been prepared using a set of Python scripts custom-written for the purpose of the research. We collected most of them in the Matrixer repository available on https://github.com/Zedelghem/matrixer.git.
The input preparation procedure was as follows. Two types of alignments were used as the basis for input generation: 1. the multiple sequence alignment of the individual domains (A and B) profiles (S4 Fig, S2 File); 2. the multiple sequence alignment of the extended core regions – omitting the least conserved N- and C- termini (S4 Fig).
Each family was aligned using three representative sequences. After that, to gain more resolution on the slipknotted family and the single domain families, we generated alignment with 10 representatives for PF03390, PF03788, PF04172 and three representative for all other families. The full alignment was then combined with a feature matrix [15]. The matrix was prepared based on the CLANS clustering analysis of the domains profiles, encoded in the standard, ten character alphabet (0-9; S11 Fig).
The extended core-only alignment was combined with the same CLANS matrix and a binarized N/C termini feature matrix. The N/C termini matrix encodes parts of the sequence missing from the extended core alignment and thus was not amplified to match the amino acid sequence of the alignment. The clustering matrix was amplified five, ten, fifteen and twenty times and we ran all the combinations to control the strength of the influence of the repeated features on the data. Also, we ran the clustering matrix alone and the full sequences alone to control the influence of the clustering on the structure of the tree.
Mr Bayes was run using the LG model selected as the best model for our dataset by ModelTest program [14, 16]. We run 10 million generation with sample frequency 1000 to obtain 10000 samples from the run. After the MrBayes run was completed, 25% of the first samples were removed for the step of summarizing the parameter values and trees. For the trees presented here the Potential Scale Reduction Factor (PSRF) was close to 1, effective sample size (ESS) was more than 200. Trees were visualized using FigTree software. All the trees presented have been calculated and presented as unrooted (Figs 5B and S5-8).

1.7 Visualization and figures preparation

Tree visualization was done in FigTree. Sequence alignments were analyzed in SeaView and BioEdit [6, 17]. Structures were analyzed in VMD and YasaraView [8, 9]. Missing loops in structures were built using GapRepaier [18]. Pictures of the structures were prepared in VMD and YasaraView [8, 9].

References

1. Jamroz, Michal and Niemyska, Wanda and Rawdon, Eric J and Stasiak, Andrzej and Millett, Kenneth C and Sułkowski, Piotr and Sulkowska, Joanna I (2014) KnotProt: a database of proteins with knots and slipknots, *Nucleic acids research* 43(D1), D306-D314.

2. Brunskill, Eric W and Bayles, Kenneth W (1996) Identification of LytSR-regulated genes from Staphylococcus aureus. *Journal of bacteriology* 178(19), 5810-5812.

3. Zimmermann, Lukas and Stephens, Andrew and Nam, Seung-Zin and Rau, David and Kübler, Jonas and Lozajic, Marko and Gabler, Felix and Söding, Johannes and Lupas, Andrei N and Alva, Vikram (2018) A completely reimplemented MPI bioinformatics toolkit with a new HHpred server at its core. *Journal of molecular biology* 430(15), 2237-2243.

4. Cao, Baoqiang and Porollo, Aleksey and Adamczak, Rafal and Jarrell, Mark and Meller, Jaroslaw (2005) Enhanced recognition of protein transmembrane domains with prediction-based structural profiles. *Bioinformatics* 22(3), 303-309.

5. Pie, J and Kim, BH and Grishin, NV (2008) PROMALS3D: a tool for multiple sequence and structure alignment. *Nucleic Acids Res* 36(7), 2295-300.

6. Galtier, Nicolas and Gouy, Manolo and Gautier, Christian (1996) SEAVIEW and PHYLO_WIN: two graphic tools for sequence alignment and molecular phylogeny. *Bioinformatics* 12(6), 543-548.

7. UniProt Consortium (2018) UniProt: a worldwide hub of protein knowledge. *Nucleic acids research* 47(D1), D506-D515.

8. William Humphrey and Andrew Dalke and Klaus Schulten (1996) VMD-Visual Molecular Dynamics. *Journal of Molecular Graphics* 14, 33-38.

9. Krieger, Elmar and Vriend, Gert (2014) YASARA View-molecular graphics for all devices—from smartphones to workstations. *Bioinformatics* 30(20), 2981-2982.

10. Frickey, Tancred and Lupas, Andrei (2004) CLANS: a Java application for visualizing protein families based on pairwise similarity. *Bioinformatics* 20(18), 3702-3704.

11. El-Gebali, Sara and Mistry, Jaina and Bateman, Alex and Eddy, Sean R and Luciani, Aurélien and Potter, Simon C and Qureshi, Matloob and Richardson, Lorna J and Salazar, Gustavo A and Smart, Alfredo and others (2018) The Pfam protein families database in 2019. *Nucleic acids research* 47(D1), D427-D432.

12. Li, Weizhong and Godzik, Adam (2006) Cd-hit: a fast program for clustering and comparing large sets of protein or nucleotide sequences. *Bioinformatics* 22(13), 1658–1659.

13. Berman, Helen M and Battistuz, Tammy and Bhat, Talapady N and Bluhm, Wolfgang F and Bourne, Philip E and Burkhardt, Kyle and Feng, Zukang and Gilliland, Gary L and Iype, Lisa and Jain, Shri and others (2002) The protein data bank. *Acta Crystallographica Section D: Biological Crystallography* 58(6), 899–907

14. Ronquist, Fredrik and Huelsenbeck, John P (2003) MrBayes 3: Bayesian phylogenetic inference under mixed models. *Bioinformatics* 19(12), 1572-1574.

15. Majorek, Karolina A and Dunin-Horkawicz, Stanislaw and Steczkiewicz, Kamil and Muszewska, Anna and Nowotny, Marcin and Ginalski, Krzysztof and Bujnicki, Janusz M (2014) The RNase H-like superfamily: new members, comparative structural analysis and evolutionary classification. *Nucleic acids research* 42(7), 4160-4179.

16. Posada, David and Buckley, Thomas R (2004) Model selection and model averaging in phylogenetics: advantages of the AIC and Bayesian approaches over likelihood ratio tests. *Syst. Biol*

17. Hall, Tom A and others (1999) BioEdit: a user-friendly biological sequence alignment editor and analysis program for Windows 95/98/NT. *Nucleic acids symposium series* 41(41), 95-98.

18. Jarmolinska, Aleksandra I and Kadlof, Michal and Dabrowski-Tumanski, Pawel and Sulkowska, Joanna I (2018) GapRepairer: a server to model a structural gap and validate it using topological analysis *Bioinformatics* 34(19), 3300-3307.

19. Steinegger, Martin and Meier, Markus and Mirdita, Milot and Voehringer, Harald and Haunsberger, Stephan J and Soeding, Johannes (2019) HH-suite3 for fast remote homology detection and deep protein annotation. *bioRxiv* 560029.

20. Potter, Simon C and Luciani, Aurélien and Eddy, Sean R and Park, Youngmi and Lopez, Rodrigo and Finn, Robert D (2018) HMMER web server: 2018 update *Nucleic acids research* 46(W1), W200-W204.

21. Chang, Abraham B and Lin, Ron and Studley, W Keith and Tran, Can V and Saier, Jr, Milton H (2004) Phylogeny as a guide to structure and function of membrane transport proteins. *Molecular membrane biology* 21(3),171-181.

22. Yang, Soo-Jin and Rice, Kelly C and Brown, Raquel J and Patton, Toni G and Liou, Linda E and Park, Yong Ho and Bayles, Kenneth W (2005) A LysR-type regulator, CidR, is required for induction of the Staphylococcus aureus cidABC operon. *Journal of bacteriology* 187(17), 5893-5900.

23. Ranjit, Dev K and Endres, Jennifer L and Bayles, Kenneth W (2011) Staphylococcus aureus CidA and LrgA proteins exhibit holin-like properties. *Journal of bacteriology* 193(10), 2468-2476.

24. Pick, Thea R and Bräutigam, Andrea and Schulz, Matthias A and Obata, Toshihiro and Fernie, Alisdair R and Weber, Andreas PM (2013) PLGG1, a plastidic glycolate glycerate transporter, is required for photorespiration and defines a unique class of metabolite transporters. *Proceedings of the National Academy of Sciences* 110(8), 3185-3190.

25. Wang, Junhui and Bayles, Kenneth W (2013) Programmed cell death in plants: lessons from bacteria? *Trends in plant science* 18(3), 133-139.

26. Hagenbuch, Bruno and Stieger, Bruno and Foguet, Montserrat and Lübbert, H and Meier, Peter J (1991) Functional expression cloning and characterization of the hepatocyte Na+/bile acid cotransport system. *Proceedings of the National Academy of Sciences* 88(23), 10629-10633.

27. da Silva, Tatiana Claro and Polli, James E and Swaan, Peter W (2013) The solute carrier family 10 (SLC10): beyond bile acid transport. *Molecular aspects of medicine* 34(2-3), 252-269.
