# Supplementary material for: Slipknotted and unknotted monovalent cation-proton antiporters evolved from a common ancestor
Source: PLoS Comput Biol. 2021 Oct 14;17(10):e1009502. doi: 10.1371/journal.pcbi.1009502 (PMC8562792; doi:10.1371/journal.pcbi.1009502)
Supplement: Attachment — Submitted filename: Reviewer_response.docx [file pcbi.1009502.s016.docx]

Dear Reviewers,

Thank you for the effort in reviewing our manuscript and for the constructive comments about our work. Below we state point-by-point response to all remarks stated (comments are in black and responses in red). In the current version we have included an additional protein from the same family with the same slipknot topology, which we found to have been recently deposited in KnotProt database.

Reviewer #1: In the manuscript “Slipknotted and unknotted monovalent cation-proton antiporters evolved from a common ancestor” by Zayats et al. the authors study the evolutionary origin of the slipknot topology in proteins. After identifying several protein families, which show (slip)knotted and unknotted topologies, their phylogenetic relationship and evolutionary history is analysed and evolutionary scenarios, leading to the slipknot topology coming from an unknotted ancestor, are inferred.
In its current form, the manuscript is unfortunately not suited to provide new insights into the origin(s) of this special slipknot topology, due to deficiencies in the presentation of the results.
The following comments about the, in my opinion, major issues and shortcomings of the manuscript hopefully help to clarify several points to make the manuscript finally comprehensible and the approach reproducible for a broader audience:

Language:
The manuscript needs excessive proof reading (preferably by a native speaker), since not only many spelling mistakes and wrong words or grammar hinder comprehension, but also lead to ambiguous meanings of sentences or render the understanding of presented results or conclusions impossible.

We asked a native speaker to proof read the manuscript.

Additionally, many used words have specific meanings in the field of evolutionary biology (e.g. pathway), which differ from the context in which they are used here. This can lead to confusion and should be revised (see also Consistency and Structure paragraphs).

We agree that this was not the best term. We rephrased some terms to better fit the context of the paper, including changing "evolutionary pathway" to "evolutionary scenario".

Consistency and definition of terms:
Many terms are not used consistently throughout the manuscript and make an understanding very difficult. Most of these terms are also never explained or just after they were used already multiple times (see also Structure paragraph). Especially the terms or IDs related to the Pfam database (Pfam, pfam, PFAM, PFam etc. all exist btw → consistency) are likely not known (with their definitions) by most people and should be explained properly to provide guidance to interpretation and comprehension of the findings. PFxxxx and CLxxxx are used for example extensively in sentences, but if I wouldn’t know that these are IDs of Pfam entries or corresponding Pfam clan IDs, respectively (which is never explained), the findings or conclusions based on this are not understandable at all. The same with the use of family/repeat/domain – I have the feeling here the HMM-types from the Pfam database are meant, but it is not common knowledge how these are defined within the Pfam database.

We enhanced the section with additional explanation, and we have rewritten parts of the text and focused on increasing the clarity. Additionally, we prepared the table (Table 1) with Pfam and clan ID’s and families names for each family from our dataset.

Especially, the use of “repeat” should be carefully re-evaluated, since it is often not clear if a (tandem-)repeated domain in a certain HMM/family/protein is meant or a single instance of that “Repeat” (type in the Pfam database).
Colour-coding in and across figures could be improved to ensure consistency as well.

We agree that the term "repeat" can be ambiguous, so we changed it to "domain" to show that it refers to a part of the protein. We changed it also across all the figures. We adjusted the colors in the main Figure 6B (background colored areas of the one-domain protein to the same shade of magenta and background color of proteins with unknown topology to gray) and added colored labels indicating domains A and B to the tree in the Figure 6B. Also, we added colored labels in the Suppl. Figures 5-8 that match colors of the main Figure 6. Additionally, we removed black lines from the Figure 6B indicating number of the domains in protein family, which was perhaps misleading in the tree built from individual domains.

Structure:
Although most information is provided somewhere in the manuscript, it takes multiple readthroughs to find the according explanations or used methods and understand afterwards the related sentences.
If the Methods are at the very end of the manuscript, some simple explanations what has been done and how, needs to go into the Results section. The Results section contains furthermore at the beginning parts that are introduction and throughout the section parts, which are Discussion. Especially the presentation of the Results should be restructured completely (with consistency and definition of terms at first appearance in mind; see above).

Thank you, after a long break from the text we also see that it could be difficult to read. Thus, we have cleaned up and moved some of the information in the methods to earlier sections of the manuscript. In particular, we merged the Results and Discussion sections and expanded the Conclusions section.

Missing information:
Some information is missing completely and should be provided to make the conclusions comprehensible and reproducible. Especially in the beginning of the Results section: where have how many sequences been taken from and analysed/how big are the analysed families/what are their properties?

We have added a table showing all 17 protein families on which the work is based. We also added some details regarding number of sequences used to the Method section.

Description and explanation of the methodology (how were the 7 evolutionary scenarios inferred?).

We have added a broader description in the result section of the evolutionary scenarios we found, with particular emphasis on how they are derived.

The gitlab repo linked in the manuscript is not existent, therefore I couldn’t have a look at the source code and see what has been done.

Thank you, we corrected the link.

In many figures information or explanation is missing (double-check also Supplementary Info, S12 e.g. does not provide any information of what is shown (headers of the columns or description and meaning of the character states) etc. etc.).

Thank you for pointing this out. We improved and expanded explanations in the legends of all of the figures, including the headers in the figure mentioned by the Reviewer.

The role of repeats (and potential tandem repeats formed by tandem duplications rather than “fusion” of two single-domain proteins) should be explained and visualised.

We acknowledge that perhaps the use of the word repeat was not the best option for our manuscript and created some misunderstanding. As the Reviewer suggested, we decided to change it to domain through the whole manuscript. Therefore, instead of the repeat 1/2 we use domain A/B. On Fig. 2 we show that a one-domain protein is a common ancestor for both two-domain slipknotted and unknotted proteins therefore showing the role of a single domain (previously repeat). In Fig. 5 we show in detail how the proteins are built from (repeated) domains A and B. In Suppl. Fig. 2 we show that in four families with known structures, proteins are composed of two inverted domains connected by the linker. Thus, Figure 5 and Suppl. fig. 2 visualize the role of domains (previously repeats) in proteins studied here.

The provided Pfam-IDs can represent HMMs for example, which span already multiple instances of one repeat. How many repeat instances are included in the single-coloured bars in the figures or the according mentioned Pfam-IDs in the text does not become clear.

Each Pfam ID represents single protein family, which are either two-domain or one-domain. Newly added Table 1 in the main text summarizes the families. Therefore, each domain A/B (previously repeat) represents the family profile, which is derived from multiple sequence alignment of the whole family.

Also, it should be clearly indicated in the text when conservation/similarity is discussed/considered from a structural or sequence perspective.

Thank you, we have added information about this to the text.

Most conclusions made in the Results section are not comprehensible (probably because the methods behind it are missing) and should be elaborated. One of the conclusions is that observable patterns are biased “due to lack of data”; has any quality assessment been conducted to estimate this bias?

We clarified and expanded the Results section. The phrase "lack of data" refers to the lack of bacterial sequences in the database, which is likely due to the undersupply of sequenced proteins rather than the true non-existence of this family in bacteria.

Finally, it should be made very clear what has been found for the first time in this study and what is actually the achievement of the authors in this study.

Thank you, we rephrased the sentences and highlighted our findings.

The text reads in its current version very cryptic regarding the contribution (identification of a “new” slipknotted family, but all that based on existent Pfam-IDs and clans and PDB structures?), which makes it hard to distinguish between discussed former findings and the findings of this study.

Our main finding is the identification of the existing protein family as slipknotted with unique conserved pattern. Although this protein has been characterized, its nontrivial topology has not been observed. Moreover, we also found that this family has unknotted homologues, thus we were able to perform phylogenetic analysis, that was not done before, that included both slipknotted and unknotted proteins.

Reviewer #2: In this manuscript, Zayats et al. investigated the evolution of a family of Slipknotted proteins, i.e., 2-hydroxy carboxylate transporter family. Importantly, they found distantly related protein families with unknotted topology and used this divergence to identify the scenarios behind the evolution of Slipknotted proteins. Overall, this is an interesting study and provides important details for the evolution of protein topology in a specific case. The manuscript, though, can benefit from a revision to enhance clarity and presentation. Here is the list of my major and minor comments:

Major comments:

1) The evolution of membrane transporters from one-domain proteins is not surprising and thoroughly discussed in the literature. I encourage authors to focus on the mechanisms of the divergence of Slipknotted and unknotted proteins and the fact that they could identify different scenarios that explain this divergence.

We agree that such perspective is better for the results presentation. Based on our data, it can be seen that unknotted proteins as they belong to many different protein families have evolved in diverse ways. In contrast, slipknotted proteins that are representatives of only one family have followed a single evolutionary pathway that is not specific to them and is also available to unknotted proteins. Thus, as pointed out by first reviewers we rewrote some parts of the paper taking into account your suggestions.

2) The major assumption behind this work is that the difference in the topology of slipknotted and unknotted proteins is caused by differences between TM helices and loops. Although this assumption sounds plausible based on previous studies (cited by the authors), authors should elaborate and justify why this assumption likely holds. They may use the current dataset and employ statistical analyses to see whether TMs and the loops contribute the most to such divergence.

Thank you for pointing it out. We added additional information in the text which show that the reason for the apparent differences in topology between proteins is the GGxG motif present in the transmembrane helices. We have added Suppl. Figure 1 showing a comparison of sequence profiles for slipknotted and unknotted proteins. From this it can be seen that this motif is only present among slipknotted proteins and it is responsible for the helix bending that promotes the formation of a non-trivial topology.

Minor comments:

1) The manuscript is well-written but can greatly benefit from the substantiation of important concepts. For example, the authors should explain why knotted proteins are interesting and cover the most important literature on this problem.

We added additional information about possible role and function of slipknotted motifs and discussed in details possible mechanisms of folding slipknotted and knotted proteins, which is one one the major challenges in the field of proteins with non-trivial topology. Also, we point out the readers to current reviews and expanded literature list.

2) Authors write "It was shown, that globular, as well as transmembrane, slipknotted proteins often appear in new organisms" in Lines 13-14. This is an overstated claim as the cited paper is only talking about the one protein family (2-Hydroxycarboxylate Transporter Family).

Thank you for pointing this out, we rewrote this statement, since the work it was based on, only contained a single family.

3) The domains and regions that were used for evolutionary analyses should be clarified in the text. The current writing can confuse the readers. For example, authors write in lines 48-49 "thus we used a single repeat from each structure for comparison". The sequence position should be stated clearly.

We agree, and we added information about sequence positions in the representative structures of slipknotted and unknotted proteins to the text. Additionally, we modified Suppl. Figure 4 showing the regions we refer to (domains, conserved core and extended core).

4) I found figures S2 and S3 (of the supplementary information) quite informative. Why don't authors bring up these figure to the main text and discuss them when they write about the superposition of knotted and unknotted proteins (Lines 48-63).

Thank you. We added the figures to the main text.

5) In constructing figures S5 and S6 and Fig. 3, it appears that the authors used the full sequence of N- and C-repeats. Would their clustering change if they only look at TMs and the connecting loops?

Thank you for pointing is out, it seems that our explanation was not sufficient. The data behind figures the Reviewer mentioned is based only on the transmembrane region, so the domains’ (previously repeat) sequences alone. Structurally unaligned helices and interdomain linkers are not included into these sequences. Therefore, for each domain we only used TMs helices and the loops that are connecting them. We added additional explanations. Also, we corrected and expanded Suppl. Figure 4 which was perhaps misleading before. Now it shows and names correctly all the regions used in our work (domain, conserved core and extended core).

Reviewer #3: The article scrutinizes evolutionary relations between unknotted and slipknotted protein domains by using the sodium-citrate transporter as an example of a protein with a slipknotted domain. The topic of the article is very interesting, and it deserves publication in PLOC Computational Biology after revision.
Before the publication, several points should be addressed.
General Comments:
The manuscript should be amended to make it understandable to the general reader of PLOS Computational Biology.
Specifically, the difference between the unknotted, knotted and slipiknotted domains should be clearly explained in the Introduction section of the main text.

Thank you. To see a clear difference between these topologies is crucial for this paper. Following Reviewer’s suggestion, we added additional explanation including figure (current Fig.1 based on Fig. S1).

The Fig. 1 is not quite successful. It does not explain how a knot is formed from a flat sequence profile shown on the top. Perhaps, intermediate steps of the knot formation should be shown. The Fig. S1 can be used in the main text to make the explanation clearer.

Thank you. We added fig. S1 (now Figure 1) to the main text. We would like to point out that slipknot can be formed at least in three different ways. We added section dedicated to folding of proteins with non-trivial topology to the Result and Discussion section.

Also, the topology of the citrate transporter should be explained in more detail. This protein consists of functionally distinct scaffold and elevator domains. From the current text it is not clear whether all the analysis is related to the whole protein or only to its elevator domain. Therefore, the overall topology of the protein should be described and shown.

Only after that, the evolutionary relation of the slipknotted (elevator?) domain to other proteins (domains?) should be analyzed in detail.

Thank you for pointing this out. Division into scaffold and elevator domains is important concept in transporters. It seems that our explanation was not the easiest to follow what was done. In this study we used whole transmembrane region, which consists of both scaffold and elevator domains - all transmembrane helices are included in our analysis. The scaffold and elevator domains are distinguished based on motions of the transporter during the transport (transition from inward to outward-open states). In contrast, here we divided the transmembrane region into repeated domains A and B which are evolutionary conserved. These repeated domains A and B are identified based on structural and sequence similarity but not on motion (as scaffold and elevator). So, both scaffold and elevator are included here, however now they are parts of domains A and B. Therefore, we do not discuss the elevator and scaffold in this work as it outside of the topic in our opinion. We hope that updated figures and expanded explanations throughout the text will help to clarify this.

Fig. S2 could be also used in the main text as an explanatory image albeit after modification. Currently there is hardly any difference between the panels S2A and S2C (left).

We modified and incorporated the figure in the main text.

Generally, the authors should imagine a biologist without any idea about slipknotted domains and write for such a reader.

We took this suggestion into account when including all modifications.

Specific Comments:
1. In the given family of transporters, the authors describe proteins with unknotted and slipknotted domains. It remains, however, unclear whether it is possible – topologically - to get to a slipknotted domain directly from an unknotted domain or the evolutionary transition should go via a knotted intermediate. Discussion of this point is highly desirable.

We have added a new paragraph discussing what the possibilities are for creating a slipknot in the structure into Results and Discussion section. This and a new Figure 1 should help the readers without topological background to understand possible folding mechanisms.

2. Line 104 and after: the complete multiple alignment with all amino acid sequences should be provided in Supplementary materials.

We provide the MSA as a Supplementary File 2.

3. Fig. 3. Something is wrong with the color code in Fig. 3B. There are colors that are not mentioned in the figure caption.

We added the missing labels to the caption of the figure.

4. Line 152: a word appears to miss after “widely”.

Corrected.

5. Line 167 and after: PDB IDs of the four known structures of relevant proteins should be explicitly given somewhere in the manuscript. The reference to Fig. 3B is not enough; neither the figure not the caption contains the PDB IDs.

We added this information.

6. The Discussion section is very brief. It might be interesting to discuss whether the slipknotting brings any functional advantages.

We added a paragraph pointing to a probable function of the slipknot loop in the protein we study. We also gave references to other publications where possible role of slipknot is discussed.

7. The discussion of PF03616, PF03601 and PF05684 should be moved from Conclusions into Discussion.

We added this section to newly merged Results and discussion section.

Have all data underlying the figures and results presented in the manuscript been provided?
Large-scale datasets should be made available via a public repository as described in the PLOS Computational Biology [data availability policy](http://journals.plos.org/ploscompbiol/s/data-availability), and numerical data that underlies graphs or summary statistics should be provided in spreadsheet form as supporting information.

Reviewer #1: No: The gitlab repo with the source code provided as a link in the manuscript does not exist.

We deposited the program on the laboratory github (https://github.com/ilbsm/HHsearch-results-aligner)

Reviewer #2: Yes

Reviewer #3: No: The multiple amino acid sequence alignment is absent

We added this file to the supplement.

We thank you for the effort made in judging our manuscript and for all the comments. We hope, that after the corrections we applied, our manuscript is suitable for high standards of Plos Computational Biology.

On behalf of all authors,

Joanna Sulkowska
