# Supplementary material for: Slipknotted and unknotted monovalent cation-proton antiporters evolved from a common ancestor
Source: PLoS Comput Biol. 2021 Oct 14;17(10):e1009502. doi: 10.1371/journal.pcbi.1009502 (PMC8562792; doi:10.1371/journal.pcbi.1009502)
Supplement: Attachment — Submitted filename: Review_response_27_08_21.docx [file pcbi.1009502.s017.docx]

Dear Reviewers,

Thank you for the effort in reviewing our manuscript and for the constructive comments about our work. Below we state point-by-point response to all remarks stated (comments are in black and responses in red).

Reviewer #2: I appreciate the authors' efforts to answer all the raised concerns. The manuscript is substantially improved and merits publication in PLoS Computational Biology. The figures' quality, however, could still be improved and I suggest authors and the editorial office to consider this issue.

We carefully evaluated the quality of the figures and improved them where necessary, we also added a new figure to improve the presentation of the potential evolutionary pathways of slipknotted membrane transporters (Fig. 6).

Reviewer #3: General comments
1. The problem with the manuscript is still that some of its sections are incomprehensibly written. Apparently, experts in topology of knots, while applying their science to proteins, have developed their own terminology, which differs from that generally accepted in protein science. Since the manuscipt deals not only with the topology but also with the evolution of a large family of membrane transporters, the article should be understandable to researchers who work on these transporters. There is no reason to expect these researchers to know the mathematical theory of knots.
The terms "entlargement", "closed knot", "open knot" and "subchain of the backbone" are unusual in protein science and should be strictly defined in the text or replaced by more traditional and understandable analogues.

Following reviewer suggestions this part is rewritten.

2. This reviewer recommends that the first paragraph of the results (or better, the whole text) be given to some biochemist to read and then edited according to the recommendations received.

The whole text was proofread by the biochemist and revised.

3. The structures shown in Figures 3A and 3C are superposed in Fig. 3B. However, the green structure from Fig. 3A is painted blue in Fig. 3B, and the blue structure from Fig. 3C is painted green in Fig. 3B. The colouring of the structures in Fig. 3B should be changed.

We merged Fig. 3 with Fig. 5 and improved the quality and color coding of the figures.

Reviewer #4: This manuscript describes a broad family of transmembrane proteins where a slipknot has been found, along with homologues that are unknotted. Much of the paper deals with ideas about gene duplication and fusion for evolution in this family. The strength of the study is that the observation is intriguing, raising interesting evolutionary and functional questions. The limitation is that whether knotting features are important in proteins remains mainly speculative. Nonetheless, the evolutionary arguments are interesting, and the findings should be useful to those interested in transmembrane transporter function and evolution.

The revisions noted appear to have been largely effective at improving the original paper. Two issues for further improvement, one regarding substance and clarity and the other regarding presentation are:

1) The topological features that distinguish a slipknot from an unknot in a protein chain are hard to grasp even for experts and certainly for newcomers. The figures provided go part way to explaining things, but my sense is that most readers will remain somewhat befuddled. I suggest that the authors augment figure 4 with a simplified curve drawing for the knotted and unknotted curves, with a focus on explaining how two structurally similar cases can be topologically different. For example, can a diagram be drawn that might show that the difference arises from one structure having a curve crossing one way while the other structure has a curve crossing the other way. A diagram like this would go along way towards drawing in more readers with a connection between the sequence/structure evolution and the topological difference being reported.

We updated the figures (current Figs. 2, 3 and 4) to better show what the slipknot is and how it is different topologically from an unknotted chain. On Fig. 2 we show schematic diagram of the slipknot and unknotted chain and on Fig. 3 we added detailed characterization of the entangled region based on the protein’s sequence and structure.

2) The overall clarity of the paper is ok, and the mechanics are generally good. But some further editing is required. Maybe this can be done safely at the technical editor level. One point to focus on is the use of commas, which are not placed well in many instances (lines 188, 220, 308, and others). The negative use of “for none of these” in line 39 should be reworded. In line 143, ‘spacial’ should be ‘spatial’. In line 315, ‘constrains’ should be ‘constraints’. Words appear to be missing in line 327.

Thank you for carefully reading the paper. We revised and corrected the text.

We thank you for the effort made in judging our manuscript and for all the comments. We hope, that after the corrections we applied, our manuscript is suitable for high standards of Plos Computational Biology.

On behalf of all authors,

Joanna Sulkowska
